# Holotoxin A_1_ Induces Apoptosis by Activating Acid Sphingomyelinase and Neutral Sphingomyelinase in K562 and Human Primary Leukemia Cells

**DOI:** 10.3390/md16040123

**Published:** 2018-04-10

**Authors:** Seong-Hoon Yun, Eun-Hye Sim, Sang-Heum Han, Jin-Yeong Han, Sung-Hyun Kim, Alexandra S. Silchenko, Valentin A. Stonik, Joo-In Park

**Affiliations:** 1 Department of Biochemistry, Dong-A University College of Medicine, Busan 49201, Korea; tpohot10@nate.com (S.-H.Y.); ehsim@dau.ac.kr (E.-H.S.); idhsh371@naver.com (S.-H.H.); 2 Department of Laboratory Medicine, Dong-A University College of Medicine, Busan 49201, Korea; jyhan@dau.ac.kr; 3 Department of Internal Medicine, Dong-A University College of Medicine, Busan 49201, Korea; kskmoon@dau.ac.kr; 4 G.B. Elyakov Pacific Institute of Bioorganic Chemistry, Far-Eastern Branch of the Russian Academy of Sciences, Vladivostok 690022, Russia; sialexandra@mail.ru (A.S.S.); stonik@piboc.dvo.ru (V.A.S.)

**Keywords:** marine triterpene glycoside, acid sphingomyelinase, neutral sphingomyelinase, apoptosis, leukemia

## Abstract

Marine triterpene glycosides are attractive candidates for the development of anticancer agents. Holotoxin A_1_ is a triterpene glycoside found in the edible sea cucumber, *Apostichopus (Stichopus) japonicus.* We previously showed that cladoloside C_2_, the 25(26)-dihydro derivative of holotoxin A_1_, induced apoptosis in human leukemia cells by activating ceramide synthase 6. Thus, we hypothesized that holotoxin A_1_, which is structurally similar to cladoloside C_2_, might induce apoptosis in human leukemia cells through the same molecular mechanism. In this paper, we compared holotoxin A_1_ and cladoloside C_2_ for killing potency and mechanism of action. We found that holotoxin A_1_ induced apoptosis more potently than cladoloside C_2_. Moreover, holotoxin A_1_-induced apoptosis in K562 cells by activating caspase-8 and caspase-3, but not by activating caspase-9. During holotoxin A_1_ induced apoptosis, acid sphingomyelinase (SMase) and neutral SMase were activated in both K562 cells and human primary leukemia cells. Specifically inhibiting acid SMase and neutral SMаse with chemical inhibitors or siRNAs significantly inhibited holotoxin A_1_–induced apoptosis. These results indicated that holotoxin A_1_ might induce apoptosis by activating acid SMase and neutral SMase. In conclusion, holotoxin A_1_ represents a potential anticancer agent for treating leukemia. Moreover, the aglycone structure of marine triterpene glycosides might affect the mechanism involved in inducing apoptosis.

## 1. Introduction

Acute myeloid leukemia (AML) is a hematologic malignancy characterized by elevated proliferation of myeloid lineage precursors and impaired differentiation of normal hematopoietic progenitor cells [1]. Despite the various cures available for most leukemias, we remain challenged by cellular resistance to anticancer agents [2]. Thus, there is an increasing need for new therapeutic agents to improve the survival rate of patients with leukemia.

It was previously reported that ceramide had tumor suppressive properties [3]. Ceramide can be generated by either ceramide synthase or sphingomyelinase (SMase) [4,5]. The SMases are classified as acid, alkaline, or neutral [6,7], based on their optimal pH [8] (Reviewed in [9]). Recent reviews have emphasized that many anticancer agents kill leukemic cells by increasing ceramide production [10,11]. Thus, discovering new agents to increase ceramide production in cancer cells might be a useful therapeutic strategy in cancer therapy.

**Figure 1:**
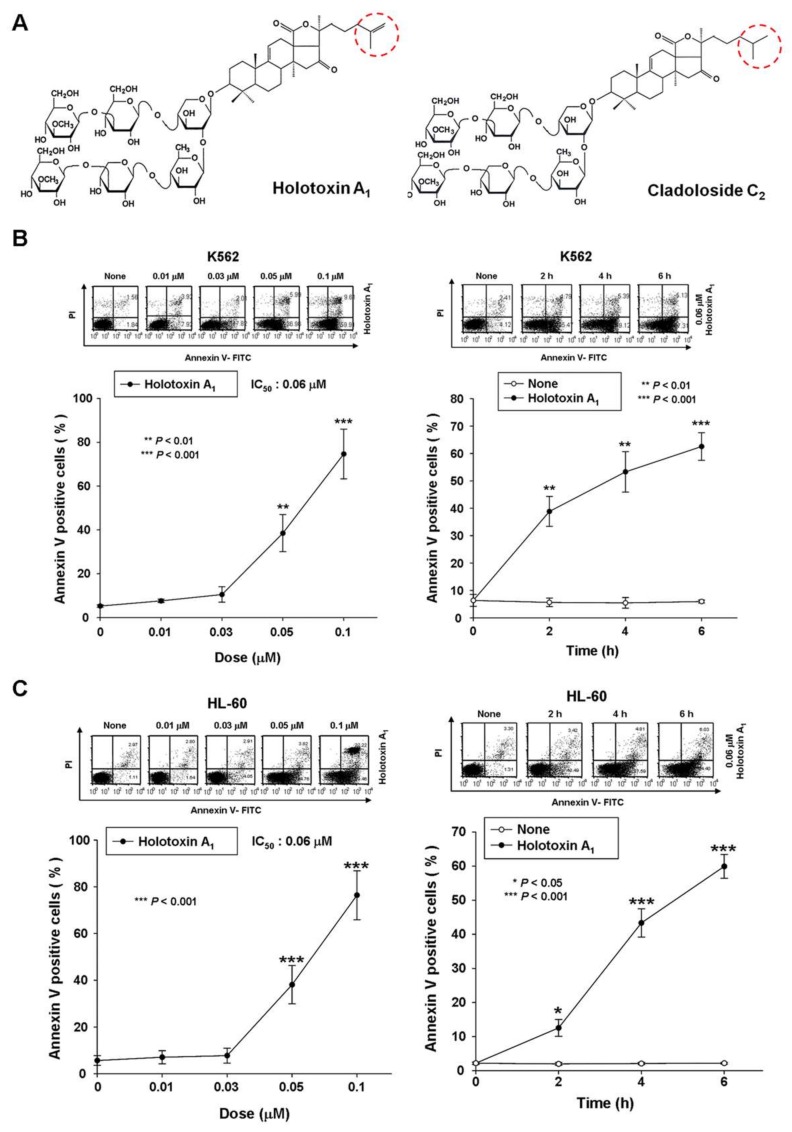

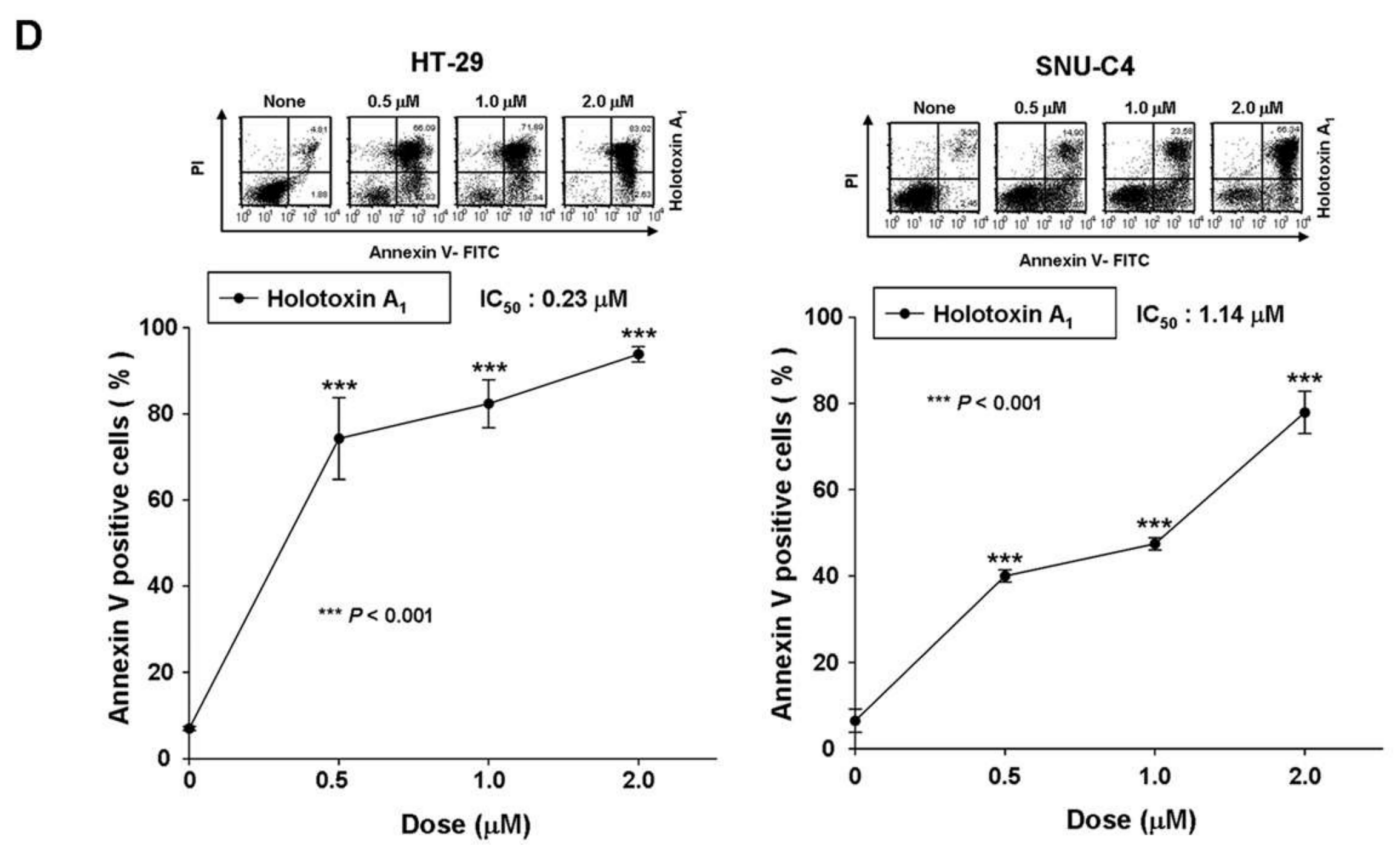
Holotoxin A_1_ induces apoptosis in human leukemic and colorectal cancer cells. (**A**) Structures of holotoxin A_1_ and cladoloside C_2_; (**B**,**C**) Cells were seeded, cultured for 4 h, and then treated with holotoxin A_1_, (left panels) for 6 h at various concentrations (0, 0.01, 0.03, 0.05, or 0.1 μM) and (right panels) for the indicated times (0.06 µM holotoxin A_1_); The percentage of apoptotic cells was determined in (**B**) K562 cells and (**C**) HL-60 cells with annexin V-FITC/PI staining; (**D**) Cells were seeded, cultured for 24 h, and then treated for 24 h with various concentrations of holotoxin A_1_ (0, 0.5, 1.0, or 2.0 μM). The percentage of apoptotic cells was measured in (left panel) SNU-C4 cells and (right panel) HT-29 cells with annexin V-FITC/PI staining; (**B**–**D**) Upper panels: Representative flow cytometry results indicate the extent of apoptosis. Lower panels: Mean ± SD of three independent experiments. * *p* < 0.05; ** *p* < 0.01; *** *p* < 0.001 vs. control cells.

Marine triterpene glycosides have various biological activities, including immunomodulatory [12], cytotoxic, antifungal, and anticancer activities [13,14]. We previously demonstrated that several marine triterpene glycosides induced apoptosis in leukemic cells, including stichoposide C (STC; a holostane glycoside with a 3β-hydroxy-23(*S*)-acetoxy-holost-7(8)-ene aglycone and six sugar units), stichoposide D (STD; a holostane glycoside with a 3β-hydroxy-23(*S*)-acetoxy-holost-7(8)-ene aglycone and one glucose instead of a quinovose in the six sugar units), and cladoloside C_2_ (a holostane glycoside with a 3β-hydroxy-16-keto-holost-9(11)-ene aglycone and six sugar units) [15,16,17]. We showed that these triterpene glycosides induced apoptosis in leukemia cells by stimulating ceramide production through different molecular mechanisms. Briefly, STC induced apoptosis in both leukemia and colorectal cancer cells by activating caspase-8, then acid SMase; the resulting depletion of glutathione caused an increase in reactive oxygen species (ROS) production, which led to neutral SMase activation [15]. In contrast, STD induced leukemia cell apoptosis by activating ceramide synthase 6 (CerS6) [16]. Finally, cladoloside C_2_ (Figure 1A) induced leukemia cell apoptosis by activating the Fas/CerS6/p38 kinase/JNK/caspase-8 pathway in lipid rafts [17]. Cladoloside C_2_ and a related marine triterpene glycoside, holotoxin A_1_, were both isolated from the holothurian, *Cladolabes schmeltzii* [18]. Of note, holotoxin A_1_ was previously found to be the main glycoside constituent of another sea cucumber, *Apostichopus (Stichopus) japonicus* [19].

Based on the fact that holotoxin A_1_ (holostane glycoside with a 16-keto-holosta-9(11),25-diene aglycone and six sugar units; Figure 1A) is a structural analogue of cladoloside C_2_, we hypothesized that holotoxin A_1_ might also induce apoptosis in leukemia cells and through the same mechanism used by cladoloside C_2_. In the present study, we tested the antitumor potential of holotoxin A_1_ in K562 cells and human primary leukemia cells, and we investigated the underlying molecular mechanisms.

## 2. Results

### 2.1. Holotoxin A_1_ Induces Apoptosis in K562 Cells by Activating the Extrinsic Pathway

To test whether holotoxin A_1_ could induce apoptosis of K562 cells, we treated cells with various concentrations of holotoxin A_1_ for different time periods and measured the extent of apoptosis with annexin V and propidium iodide (PI) staining. Holotoxin A_1_ treatment caused apoptosis, and the proportions of apoptotic cells increased in a dose- and time-dependent manner (Figure 1B). The IC_50_ of holotoxin A_1_ was 0.06 μM, much lower than that of cladoloside C_2_ (IC_50_: 0.2 μM). This finding indicated that holotoxin A_1_ was more potent than cladoloside C_2_ for inducing K562 cell apoptosis. Next, we evaluated whether holotoxin A_1_-induced apoptosis was specific to K562 cells. We performed the same experiment in other cancer cell lines, and we found that holotoxin A_1_ also induced apoptosis, but the IC_50_ of holotoxin A_1_ was different in each cell line (Figure 1C,D).

**Figure 2:**
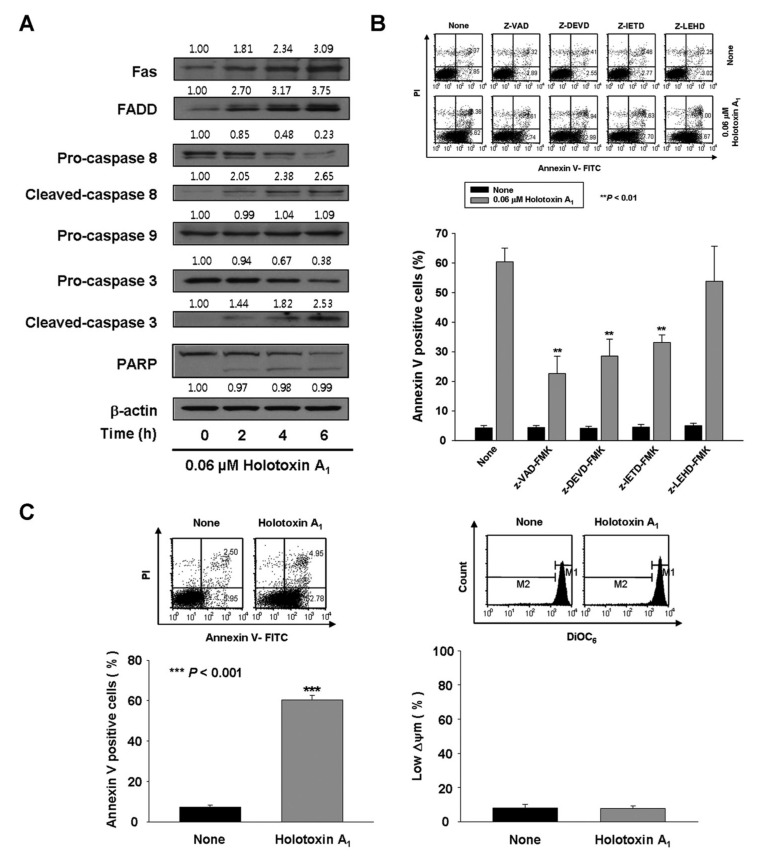

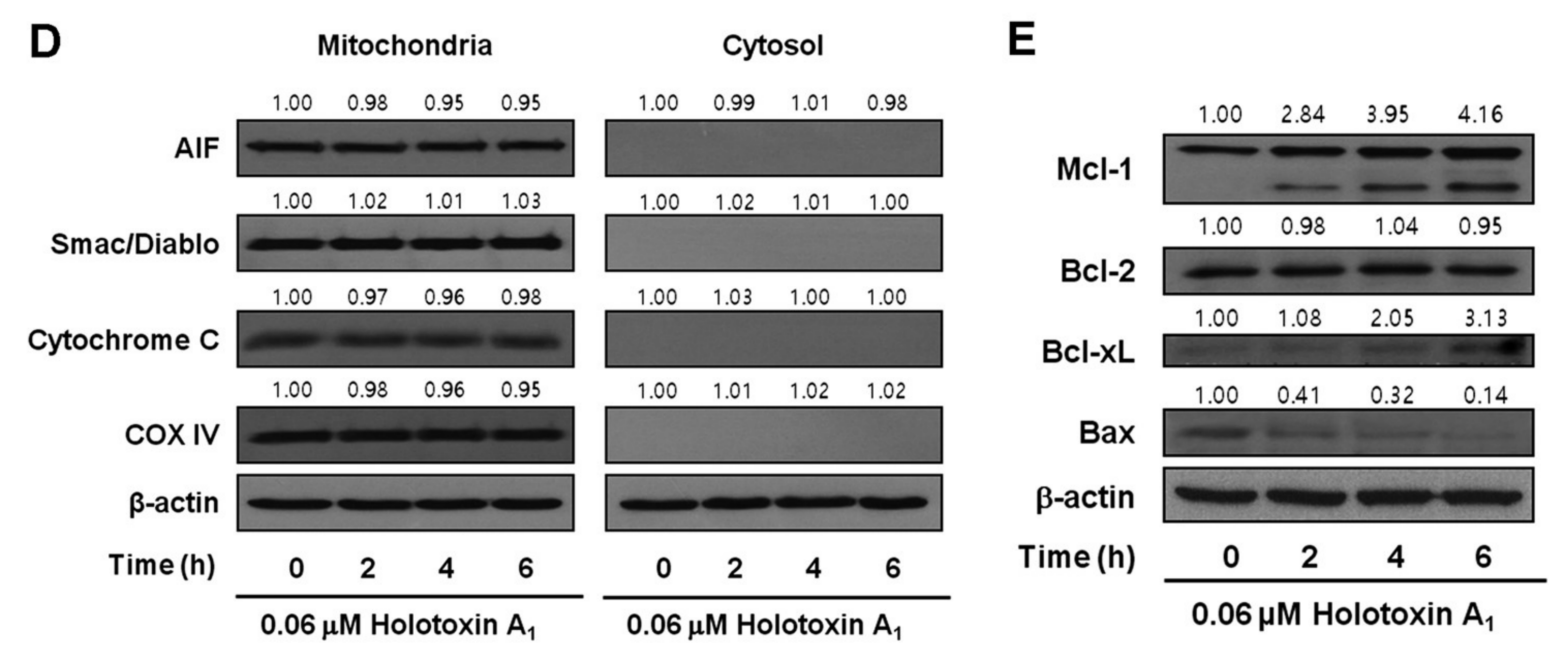
Holotoxin A_1_ induces apoptosis through extrinsic pathway activation in human leukemic cells. (**A**) Analysis of the mechanism underlying apoptosis. Western blot of K562 cell proteins after treating cells with 0.06 μM holotoxin A_1_ shows changes in protein levels over time. β-actin served as a loading control. This blot is representative of three separate experiments. Densitometry values above the bands indicate the fractional changes in protein levels, compared to initial levels at time 0; (**B**) Functional involvement of caspases in holotoxin A_1_-induced apoptosis in K562 cells. Cells were pretreated for 1 h with the pan-caspase inhibitor Z-VAD-FMK (25 μM), the caspase-8 inhibitor Z-IETD-FMK (20 μM), the caspase-9 inhibitor Z-LEHD-FMK (20 μM), or the caspase-3 inhibitor Z-DEVD-FMK (50 μM), followed by treatment with 0.06 μM holotoxin A_1_ for 6 h. (Upper panel) Representative flow cytometry results indicate the extent of apoptosis (Lower panel) The mean ± SD of three independent experiments. ** *p* < 0.01 vs. holotoxin A_1_-treated cells; (**C**,**D**) Analysis of the intrinsic pathway. (**C**) Mitochondrial membrane potential was not affected by holotoxin A_1_. Left panel: K562 cells were treated with 0.06 μM holotoxin A_1_ for 6 h. (Upper panel) Representative flow cytometry results indicate the extent of apoptosis. (Lower panel) The mean ± SD of three independent experiments. *** *p* < 0.001 vs. control cells. Right panel: K562 cells were treated with 0.06 μM holotoxin A_1_ for 2 h. The cells were stained with DiOC_6_ (3,3′_-_dihexyloxacarbocyanine iodide). (Upper panel) Flow cytometry results show mitochondrial membrane potential (∆ϕ_m_), determined by monitoring the DiOC_6_ uptake. (Lower panel) The mean ± SD of three independent DiOC_6_ uptake experiments; (**D**) Western blots show the effect of holotoxin A_1_ over time on the levels of mitochondrial (left) and cytosolic (right) proteins: AIF, Smac/DIABLO, cytochrome oxidase IV, and cytochrome c, in K562 cells. Cytochrome oxidase IV (COX IV) served as a mitochondrial marker. β-actin served as a loading control. These blots are representative of three separate experiments. Densitometry values above the bands indicate fractional changes from initial values at time 0; (**E**) Western blot shows the effect of holotoxin A_1_ over time on the levels of antiapoptotic proteins, Mcl-1, Bcl-2, and Bcl-xL, and proapoptotic protein, Bax, in K562 cells. β-actin served as a loading control. This blot is representative of three separate experiments. Densitometry values above the bands indicate fractional changes from initial values at time 0.

We also evaluated the mechanisms involved in holotoxin A_1_-induced apoptosis in K562 cells. We found that holotoxin A_1_ treatment resulted in the appearance of cleaved caspase-3 and caspase-8 (Figure 2A), which indicated that caspase-3 and caspase-8 had been activated. To determine whether caspase activation played a role in holotoxin A_1_-induced apoptosis, we performed similar experiments, but added the pan-caspase inhibitor (Z-VAD-FMK) or specific inhibitors of caspase-3 (Z-DEVD-FMK), caspase-8 (Z-IETD-FMK), or caspase-9 (Z-LEHD-FMK). We found that the induction of apoptosis by holotoxin A_1_ was significantly inhibited when cells were pretreated with Z-VAD-FMK, Z-DEVD-FMK, or Z-IETD-FMK, but not with Z-LEHD-FMK (Figure 2B). These data suggested that holotoxin A_1_ induced apoptosis through a caspase-dependent mechanism in an extrinsic pathway in K562 cells.

To investigate intrinsic pathway activation by holotoxin A_1_, we measured the mitochondrial membrane potential (MMP) and we examined mitochondrial protein release with Western blot analysis. Holotoxin A_1_-treated K562 cells showed no loss of MMP (Figure 2C) and no cytoplasmic release of cytochrome c, Second mitochondrial-derived activator of caspases (Smac)/Direct inhibitor of apoptosis protein binding protein with low pI (DIABLO), or Apoptosis-inducing factor (AIF) (Figure 2D). These results indicated that holotoxin A_1_ treatment in K562 cells activated extrinsic apoptotic pathways, and not intrinsic pathways. We next examined how holotoxin A_1_ treatment affected the levels of several antiapoptotic proteins, including B-cell lymphoma-2 (Bcl-2), B-cell lymphoma extra-large (Bcl-xL), and myeloid cell leukemia-1 (Mcl-1) and the proapoptotic protein, Bcl-2-associated X protein (Bax). The results showed that holotoxin A_1_ treatment caused increases in the expression levels of Bcl-xL and Mcl-1, a reduction in Bax expression, and did not change Bcl-2 expression (Figure 2E). The reduced expression of Bax and the elevated expression of Bcl-xL and Mcl-1 might have contributed to preserving the mitochondria.

### 2.2. Holotoxin A_1_ Induces Apoptosis in K562 Cells by Activating Fas, then Acid SMase and Neutral SMase

**Figure 3:**
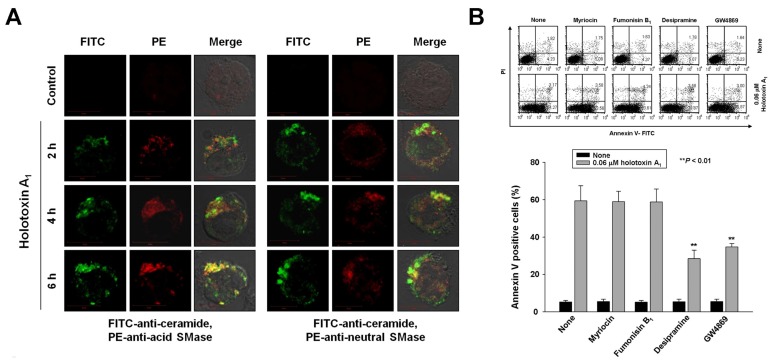

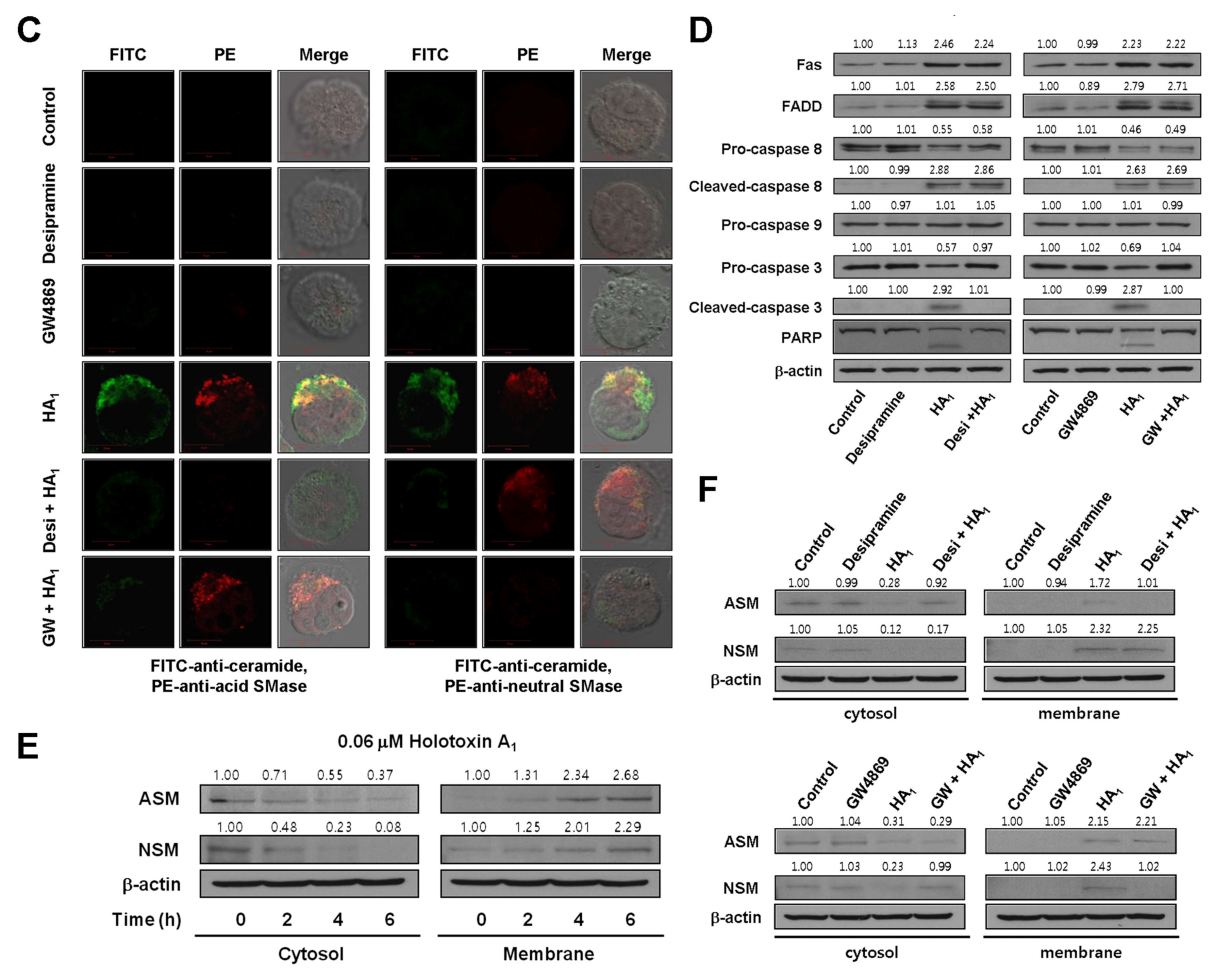
Holotoxin A_1_ induces apoptosis through activation of acid SMase and neutral SMase in human leukemic cells. (**A**) Immunohistochemistry images of K562 cells show that holotoxin A_1_ stimulated the production of ceramide (green) by activating (left) acid SMase (red) and (right) neutral SMase (red); (**B**) SMase inhibitors block apoptosis. K562 cells (1 × 10^5^ cells/well) were incubated for 6 h with holotoxin A_1_ in the presence or absence of myriocin, fumonisin B_1_, desipramine, or GW4869, and the percentage of apoptotic cells was determined with annexin V-FITC/PI staining. Upper panel: Representative flow cytometry results of three experiments for each condition. Lower panel: Mean ± SD of three independent experiments. ** *p* < 0.01 vs. holotoxin A_1_-treated cells; (**C**,**D**) Pathway analysis. K562 cells were untreated (control) or exposed to 0.06 μM holotoxin A_1_ (HA_1_) for 2 h in the presence or absence of desipramine or GW4869. (**C**) Cells were fixed, permeabilized, and then stained with FITC-labeled ceramide antibody (green) and PE-conjugated antibodies against (left) acid SMase (red) or (right) neutral SMase (red). Images are representative of three separate experiments; (**D**) K562 cell lysates were analyzed on Western blots probed with the indicated antibodies. Western blots are representative of three separate experiments. β-actin was used as a loading control. Densitometry values above the bands indicate fractional changes compared to control; (**E**,**F**) Membrane translocation. K562 cells were treated with 0.06 μM holotoxin A_1_, then cells were fractionated to analyze membrane and cytosolic proteins. (**E**) After the indicated treatment times, (left) cytosolic and (right) membrane fractions were run on western blots and probed with antibodies against acid SMase (ASM) or neutral SMase (NSM). Western blots are representative of three separate experiments. β-actin was used as a loading control. Densitometry values above the bands indicate fractional changes from initial values at time 0; (**F**) K562 cells were untreated (control) or incubated for 2 h with holotoxin A_1_ in the presence or absence of (top panels) desipramine or (bottom panels) GW4869. Western blots show (left) cytosolic and (right) membrane fractions probed with the indicated antibodies. Western blots are representative of three separate experiments. β-actin was used as a loading control. Densitometry values above the bands indicate fractional changes compared to control.

Previously, we reported that cladoloside C_2_ induced apoptosis by activating ceramide synthase 6 (CerS6) to generate ceramide [17]. Accordingly, due to their similar structures, we expected that holotoxin A_1_ would induce apoptosis through the same mechanism. To test this hypothesis, we performed immunofluorescence staining and found that holotoxin A_1_ treatment increased ceramide production in K562 cells in a time-dependent manner (Figure 3A). Next, we investigated which of three enzymes was activated by holotoxin A_1_ to induce apoptosis: acid SMase, neutral SMase, or ceramide synthase. We pretreated the cells for 1 h with desipramine (acid SMase inhibitor), GW4869 (neutral SMase inhibitor), myriocin (serine palmitoyl transferase inhibitor), or fumonisin B_1_ (ceramide synthase inhibitor), followed by holotoxin A_1_ treatment. We found that holotoxin A_1_-induced apoptosis was partially blocked by pretreatment with desipramine or GW4869, but not by pretreatment with myriocin or fumonisin B_1_ (Figure 3B). The holotoxin A_1_-mediated activation of caspase-3 was blocked by pretreatment with desipramine or GW4869; however, its activation of Fas and caspase-8 was not inhibited by pretreatment with desipramine or GW4869 (Figure 3C). Holotoxin A_1_-mediated ceramide production was also inhibited by pretreatment with desipramine or GW4869 (Figure 3D). We next investigated whether holotoxin A_1_ treatment caused a change in the subcellular location of acid SMase or neutral SMase with immunofluorescence microscopy. We observed that the holotoxin A_1_-mediated activation of acid SMase or neutral SMase resulted in translocation of the enzymes to the plasma membrane (Figure 3A,D). These translocations into the membrane from the cytosol were confirmed with Western blot analysis (Figure 3E). Moreover, the translocation of acid SMase or neutral SMase from the cytosol to the membrane was inhibited by desipramine or GW4869, respectively (Figure 3F). Taken together, these results suggested that the ceramide generated by holotoxin A_1_ activation of acid SMase and neutral SMase, might partly contribute to apoptosis induced by holotoxin A_1_ in leukemic cells.

**Figure 4:**
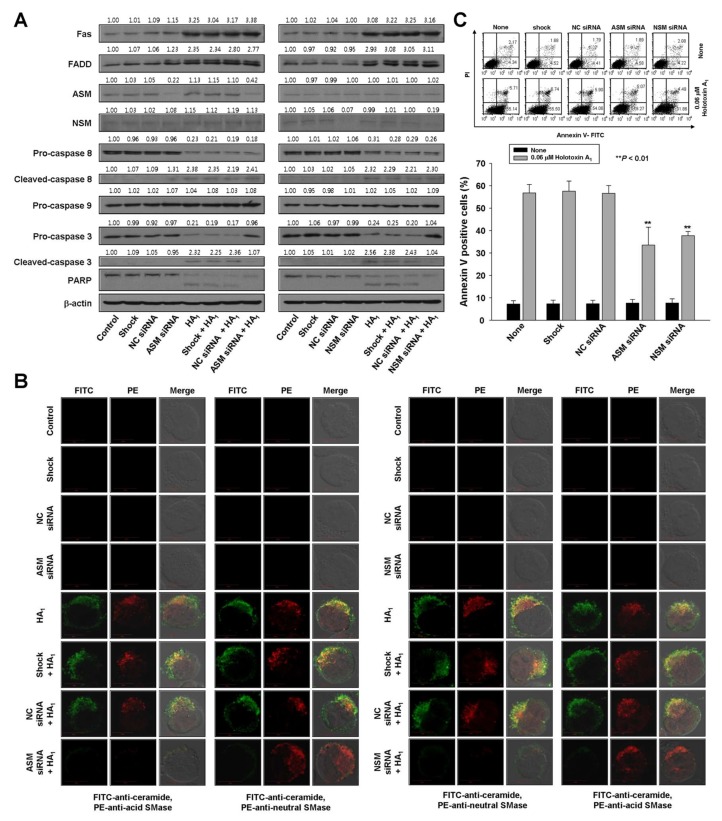
Acid SMase and neutral SMase knockdowns inhibit holotoxin A_1_-induced apoptosis in K562 cells. (**A**–**C**) K562 cells were transiently transfected by electroporation for 48 h with siRNAs against acid SMase (ASM) or neutral SMase (NSM). A nonspecific control (NC) siRNA and no siRNA (shock) served as controls. Then, cells were untreated (control) or treated with holotoxin A_1_ (HA_1_). (**A**) siRNA SMase silencing. Western blot analyses of cell lysates show pathway proteins stimulated by holotoxin A_1_ treatment in (left) cells with normal or knocked down ASM expression and (right) cells with normal or knocked down NSM expression; (**B**) siRNA SMase silencing blocks ceramide production. K562 cells transfected with (left) ASM siRNAs or (right) NSM siRNAs were treated with holotoxin A_1_ for 2 h, and then fixed and permeabilized. The samples were then stained with PE-conjugated (red) antibodies against acid SMase (three left columns), or neutral SMase (three right columns) and FITC-labeled ceramide antibody (green). Images are representative of three separate experiments; (**C**) siRNA SMase silencing inhibits apoptosis. Cells were untreated (control, shocked) or treated with NC, ASM, or NSM siRNAs and then incubated for 6 h with or without holotoxin A_1_. Upper panels: Representative flow cytometry results show the percentages of apoptotic cells determined with annexin V-FITC/PI staining. Lower panels: Mean ± SD of three independent experiments. ** *p* < 0.01, cells treated with holotoxin A_1_ versus cells transfected with acid SMase siRNA or neutral SMase siRNA and treated with holotoxin A_1_.

To verify the essential role of acid SMase and neutral SMase activation in holotoxin A_1_-mediated apoptosis, we transfected K562 cells with small interfering RNAs (siRNAs) designed to knock down acid SMase or neutral SMase expression. The control was a nonspecific siRNA. Acid SMase and neutral SMase knockdowns were confirmed with Western blot analyses and immunofluorescence staining (Figure 4A,B). The transfected cells were treated with holotoxin A_1_ and the proportion of apoptotic cells was examined. The siRNA-mediated knockdown of either acid SMase or neutral SMase protected cells from holotoxin A_1_-induced apoptosis (Figure 4C).

To investigate the sequence of events that led to holotoxin A_1_-induced apoptosis, we evaluated holotoxin A_1_-induced activation of Fas, caspase-8, and caspase-3 in K562 cells transfected with siRNAs against acid SMase and neutral SMase. The siRNA silencing reversed the activation of caspase-3, but not Fas or caspase-8 (Figure 4A). These data indicated that the holotoxin A_1_-induced activation of acid SMase and neutral SMase occurred downstream of Fas and caspase-8 activation and upstream of caspase-3 activation.

### 2.3. Holotoxin A_1_ Induces Apoptosis via Activation of Acid and Neutral SMases in Primary Human Leukemia Cells, But not in Normal Human Hematopoietic Progenitor Cells (CD34^+^ Cells)

**Figure 5:**
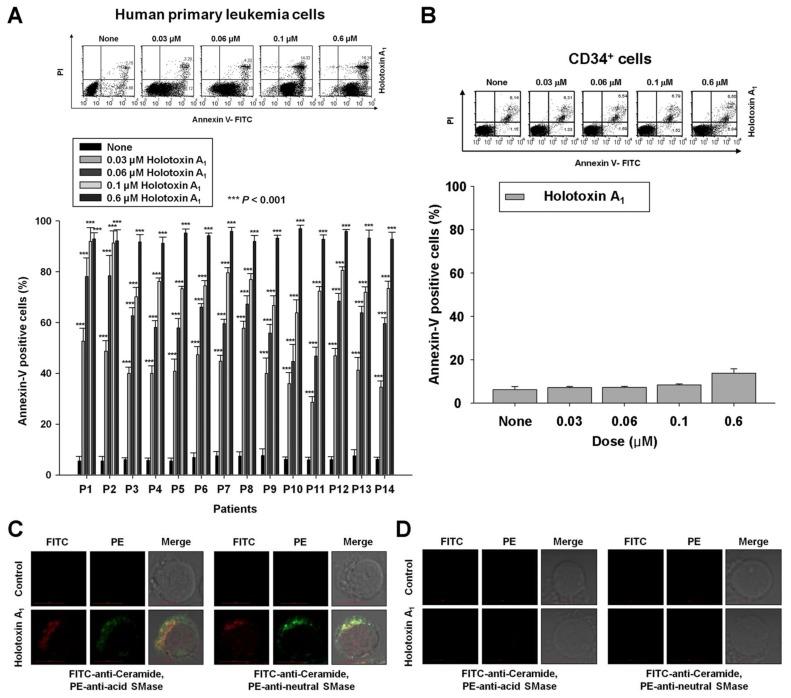
Holotoxin A_1_ activations of acid SMase and neutral SMase induce apoptosis in human primary leukemia cells. (**A**,**B**) Holotoxin A_1_ (6 h) induced apoptosis in (**A**) human primary leukemia cells, but not in (**B**) human hematopoietic progenitor CD34^+^ cells. Upper panels: Representative flow cytometry results show the percentage of apoptotic cells measured with annexin V-FITC/PI staining. Lower panels: Mean ± SD of three independent experiments. *** *p* < 0.001 versus holotoxin A_1_-treated cells; (**C**,**D**) Immunohistochemistry images show that holotoxin A_1_ (2 h) stimulated the production of ceramide (green) and activated (three left columns) acid SMase (red) and (three right columns) neutral SMase (red) in (**C**) human primary leukemia cells, but not in (**D**) human hematopoietic progenitor CD34^+^ cells. Images are representative of three separate experiments.

To investigate whether apoptosis induced by holotoxin A_1_-mediated activation of acid SMase and neutral SMase was specific to K562 cells, we performed the same experiments in other types of human primary leukemia cells. We observed holotoxin A_1_-induced apoptosis in human primary leukemia cells from 14 patients with different types of leukemia (Table 1, Figure 5A). Table 1 shows the potency (IC_50_) of holotoxin A_1_ in each cell type. These results indicated that holotoxin A_1_ induced apoptosis in multiple leukemia types. Moreover, the holotoxin A_1_ concentrations used in this study (0.01–0.6 μM) did not alter the rate of apoptosis in normal human hematopoietic progenitor cells (CD34^+^ cells), demonstrated with annexin-V/PI staining (Figure 5B).

As observed in the leukemia cell lines, we found that the molecular mechanisms underlying holotoxin A_1_-mediated apoptosis in human primary leukemia cells involved the activation of acid SMase and neutral SMase to generate ceramide (Figure 5C). Again, this holotoxin A_1_ treatment did not activate these molecules or generate ceramide in CD34^+^ cells (Figure 5D).

## 3. Discussion

Recently, an increasing number of studies have supported the notion that marine triterpene glycosides represent a potential source of therapeutically useful compounds [20,21]. The present study showed that, although the same carbohydrate chain structure is found in holotoxin A_1_ (holostane glycoside with 3β-hydroxy-16-keto-holosta-9(11),25-diene aglycone and six sugar units), STC (an acetylated triterpene glycoside with an acetoxy moiety linked to C-23 in a 3β-hydroxyholost-7(8)-ene aglycone and six sugar units) and cladoloside C_2_ (holostane glycoside with 3β-hydroxy-16-keto-holost-9(11)-ene aglycone and six sugar units, but with a saturated side chain, in contrast to holotoxin A_1_), holotoxin A_1_ was more potent than both STC and cladoloside C_2_. Based on our observations, despite the greater apoptotic potency of holotoxin A_1_ (aglycone with double bond at C-25) over cladoloside C_2_ (aglycone without double bond at C-25) both triterpenes activated the extrinsic apoptotic pathway, unlike STC, which activates the intrinsic pathway. Moreover, when we treated cells with a caspase-8 inhibitor before the holotoxin A_1_ treatment, the holotoxin A_1_-induced apoptosis was partially blocked. These data indicated that holotoxin A_1_ induced apoptosis through a caspase-8-dependent pathway. Interestingly, holotoxin A_1_ did not seem to affect mitochondria, demonstrated by the intact MMP and the absence of cytochrome c, Smac/DIBLO, or AIF release into the cytosol. In addition, we observed elevated Bcl-xL and Mcl-1 expression and reduced Bax expression. In many cancer cells, overexpression of prosurvival factors, such as Bcl-2, Bcl-xL, and Mcl-1, contributes to resistance to anticancer agents. Thus, these prosurvival proteins have become important targets for developing new anticancer drugs [22,23,24]. However, we observed that holotoxin A_1_ induced apoptosis in leukemic cells, despite the elevated expression of Bcl-xL and Mcl-1. Thus, holotoxin A_1_ might be very effective for killing cancer cells that overexpress Bcl-xL and Mcl-1. Because holotoxin A_1_ and cladoloside C_2_ share the same glycone structure, we expected that holotoxin A_1_, like cladoloside C_2_, would induce apoptosis by activating CerS6. Unexpectedly, we found that holotoxin A_1_ induced apoptosis by activating acid SMase and neutral SMase, similar to the STC mechanism of action. Holotoxin A_1_-mediated activations of Fas and caspase-8 were not inhibited when siRNAs silenced acid SMase or neutral SMase. In contrast, caspase-3 activation and ceramide production were inhibited by silencing acid SMase or neutral SMase with siRNAs. These results suggested that holotoxin A_1_ led to Fas activation, followed by the activation of caspase-8 and acid SMase. Our previous study demonstrated that STC treatment led to glutathione depletion, increased ROS production, and neutral SMase activation [15]. However, in this study, we did not investigate whether glutathione and ROS were involved in the holotoxin A_1_-mediated activation of neutral SMase. Future studies are needed to examine the detailed molecular mechanisms underlying holotoxin A_1_-induced neutral SMase activation.

Previous studies demonstrated that the biological effects of triterpene glycosides were influenced by both the aglycone and the carbohydrate chains [20,25,26]. It was known that the presence of acetoxy groups could enhance cytotoxic potency [27]. Interestingly, although holotoxin A_1_ does not have an acetoxy group, its cytotoxic effects were more potent in killing leukemia cells than STC, which contains an acetoxy group. These data indicated that the presence of keto group at C-16 of aglycone was very important for anti-leukemic activity. Our studies have also suggested that both glycone and aglycone groups, separately and in combination, were important for the biological activities of triterpene glycosides. However, their structure-activity relationships appeared to be more complicated than expected. Thus, more extensive structure-activity relationship studies of triterpene glycosides would be informative in developing new anticancer agents. Future studies are needed to investigate the antitumor activity of holotoxin A_1_ in other types of leukemia, including chemotherapy-resistant leukemia cells, and in other types of cancer. In addition, further investigation is needed to discern other molecular mechanisms that might contribute to holotoxin A_1_-induced apoptosis.

In conclusion, this study provided the first evidence that holotoxin A_1_ could induce apoptosis in K562 and human primary leukemic cells by activating acid SMase and neutral SMase. Our findings suggested that holotoxin A_1_ may be a useful candidate in developing treatments for human leukemias that feature overexpression of Bcl-xL and Mcl-1.

## 4. Materials and Methods

### 4.1. Cell Culture

Two human leukemic cell lines, K562 and HL-60, and two human colorectal cancer cell lines, SNU-C4 and HT-29, were obtained from the Korean Cell Line Bank (Seoul National University, Seoul, Korea). All cells were cultured in RPMI1640 or Dulbecco’s Modified Eagle’s Medium supplemented with 10% fetal bovine serum (FBS), 100 U/mL penicillin, and 100 μg/mL streptomycin. Human hematopoietic progenitor CD34^+^ cells were obtained from STEM CELL Technologies (Vancouver, BC, Canada), and cultured in Hematopoietic Progenitor Expansion Medium DXF with cytokine mix E (PromoCell, Heidelberg, Germany).

### 4.2. Reagents

Holotoxin A_1_ was isolated and purified with the procedure described by Silchenko et al. [18], then dissolved in sterilized distilled water. Annexin V was obtained from BD Biosciences Clontech (Palo Alto, CA, USA). Anti-Fas, anti-FADD, anti-procaspase-8, anti-cleaved caspase-8, anti-procaspase-3, anti-cleaved caspase-3, anti-procaspase-9, anti-cytochrome c, anti-Bcl-2, anti-Bcl-xL, anti-Mcl-1, anti-acid SMase, anti-neutral SMase, and anti-β-actin antibodies were purchased from Santa Cruz Biotechnology (Santa Cruz, CA, USA). Anti-poly (ADP-ribose) polymerase (PARP) antibody was purchased from Cell Signaling Technology (Beverly, MA, USA). Unless otherwise stated, all other chemicals were obtained from Sigma (St. Louis, MO, USA).

### 4.3. Apoptosis Analysis

The percentage of apoptotic cells was measured with annexin V-FITC and flow cytometry, as previously described [27]. Briefly, cells were harvested, washed with PBS (pH 7.4), centrifuged, and stained with annexin V-FITC (Pharmingen) and 2 μg/mL PI in binding buffer (10 mM Hepes, pH 7.4, 140 mM NaCl, 2.5 mM CaCl_2_) for 15 min at 37 °C in the dark. The samples were analyzed with flow cytometry, performed with a FACScan flow cytometer (BD Bioscience, Heidelberg, Germany). Data were analyzed with CellQuest software (Becton-Dickson, San Jose, CA, USA).

### 4.4. Measurement of MMP

Changes in MMP (∆ϕ_m_) were examined with DiOC_6_ (Molecular Probes, Eugene, OR, USA), as previously described [27]. Briefly, cells were treated with sterilized water or with holotoxin A_1_ for the indicated times, then they were incubated with DiOC_6_ (40 nM) for 20 min at 37 °C. Then, cells were washed and analyzed with flow cytometry. Finally, the percentage of cells with low MMP was calculated. For each sample, 10^4^ cells were investigated, and all experiments were performed in triplicate.

### 4.5. Separation of the Mitochondrial and Cytosolic Proteins

Cells were treated with sterilized water or with holotoxin A_1_ for the indicated times, then the mitochondrial and cytosolic fractions were separated as previously described [28,29]. Briefly, cells were harvested and resuspended in mitochondrial isolation buffer (20 mM Hepes-KOH, pH 7.5, 210 mM sucrose, 70 mM mannitol, 1 mM EDTA, 1 mM DTT, 1.5 mM MgCl_2_, 10 mM KCl) and protease inhibitor cocktail (Boehringer Mannheim, Mannheim, Germany) supplemented with 10 μM digitonin. Suspensions were incubated at 37 °C for 5 min and centrifuged at 12,000× *g* for 15 min. The supernatant (cytosolic fraction) was collected for Western blotting.

### 4.6. Separation of the Cytosolic and Membrane Proteins

Cells were treated with sterilized water or with holotoxin A_1_ for the indicated times. Membrane and cytosolic proteins were extracted and separated with the ProteoJET membrane protein extraction kit (Fermentas, Glen Burnie, MD, USA), according to manufacturer’s instructions. Briefly, cells (5 × 10^6^) were harvested by centrifugation for 5 min at 250× *g*, resuspended in 3 mL of ice-cold cell wash solution and re-centrifuged. Ice-cold cell permeabilization buffer (1.5 mL) was added, and the mixture was incubated for 10 min at 4 °C with continuous rocking. The supernatant (cytoplasmic protein extract) was collected for Western blotting. The pellets were resuspended in ice-cold membrane extraction buffer and incubated for 30 min at 4 °C in the thermomixer, shaking at 1400 rpm. Then, the suspension was centrifuged at 16,000× *g* for 15 min at 4 °C. The supernatant (membrane protein fraction) was used for Western blotting.

### 4.7. Western Blot Analysis

Cell lysis and Western blot analyses were performed as described previously [27]. Briefly, cells were harvested, washed with PBS, and treated with lysis buffer (20 mM Tris, pH 8.0, 137 mM NaCl, 10% glycerol, 1% Nonidet P-40, 10 mM EDTA, 100 mM NaF, 1 mM phenylmethylsulfonyl fluoride, and 10 mg/mL leupeptin). The lysates were centrifuged at 13,000 rpm for 15 min, and the concentration of protein in each lysate was determined with Bio-Rad Protein Assay Reagent (Bio-Rad Lab., Richmond, CA, USA), according to the manufacturer’s suggested procedure. The lysate samples (30 μg protein each) were separated with SDS-PAGE on an 8%, 10%, or 12% polyacrylamide gel. After electrophoresis, the separated proteins were transferred to nitrocellulose membranes (Amersham Life Science, Inc., Piscataway, NJ, USA). Blots were blocked overnight in 5% skim milk in PBS at 4 °C. Then, blots were probed with the appropriate primary antibody for 1 h. After washing, blots were probed with secondary antibody for 2 h. After another wash, the signals were detected with ECL detection reagents (Amersham, Buckinghamshire, UK), according to the manufacturer’s instructions. The blots were also probed with a monoclonal anti-β-actin antibody, which served as an internal control (Sigma, St. Louis, MO, USA).

### 4.8. Immunofluorescence Staining

Immunofluorescence staining was performed as described previously [15]. Briefly, cells were fixed and permeabilized with 1% formaldehyde/methanol in PBS for 10 min at room temperature. After that, the cells were washed, and antibodies were used, as indicated, followed by staining with FITC- or PE-conjugated goat-anti-mouse or anti-rabbit IgG (Calbiochem, San Diego, CA, USA). Then, the samples were mounted with glycerol, and analyzed with a confocal microscope (Carl Zeiss LSM 700; Carl Zeiss, Thornwood, NY, USA) equipped with a 40× C-Apochromat objective. As a negative control, cells were treated the same, but primary antibodies were omitted

### 4.9. siRNA Transfection

We purchased pre-designed siRNAs that targeted either the human acid SMase mRNA (catalog number SI00011557; ID 6609) or the neutral SMase mRNA (catalog number SI02655114; ID 6610) and the AllStars negative control siRNA (catalog number 1027280) from Qiagen (Hilden, Germany). SiRNA transfections were performed as described previously [15]. Briefly, for transfection, cells (1.3 × 10^7^ cells/0.5 mL) were resuspended in PBS and mixed with 200 nM anti-acid SMase siRNA, anti-neutral SMase siRNA, or non-silencing siRNA. This mixture was added to an electroporation cuvette with a 0.4 cm electrode gap and subjected to 300 V and 950 μF in a Gene Pulser Xcell Electroporation System (Bio-Rad, Richmond, CA, USA). After electroporation, the cells were cultured for 48 h in RPMI1640 supplemented with 10% FBS, then treated with sterilized water or holotoxin A_1_ for the indicated times. Finally, the cells were analyzed with annexin-V staining (Section 4.3), immunofluorescence (Section 4.8), and Western blot (Section 4.7) methods.

### 4.10. Statistical Analysis

Statistical analyses were performed with the SPSS 21.0 statistical package for Windows (SPSS, Chicago, IL, USA). Data are expressed as the mean ± standard deviation (SD). One-way ANOVAs were used to evaluate significant differences in cell viability between holotoxin A_1_-treated and control cells.

## Figures and Tables

**Table 1:** Characteristics of patients with different types of leukemia.

Patients	Sex/Age	Diagnosis (FAB)	Conventional Karyotype Analysis	IC_50_ of Holotoxin A_1_ (μM)
P1	F/63	AML M2	Normal female chromosome	0.013
P2	M/51	AML M3	Abnormal male chromosome 46,XY,t(15;17)(q24.1;q21.1) [26]	0.081
P3	F/37	AML M2	Normal female chromosome	0.068
P4	F/70	AML M4E	Abnormal female chromosome 46,XX,inv(16)(p13.1q22) [24]/46,XX [1]	0.058
P5	M/86	AML M4	Normal male chromosome	0.061
P6	F/53	AML M1	Normal female chromosome	0.051
P7	F/33	AML M4	Abnormal female chromosome 46,XX,t(11;19)(p15;p12) [26]/46,XX [1]	0.059
P8	M/72	AML M5b	Abnormal male chromosome 46,XY,t(6;11)(q27;q23) [26]	0.030
P9	M/79	AML M1	Abnormal male chromosome 47,XY,+4 [12]/46,XY [14]	0.076
P10	M/28	CML	Abnormal male chromosome 46,XY,t(9;22)(q34;q11.2) [25]	0.096
P11	F/40	CML	Abnormal female chromosome 46,XX,t(9;22)(q34;q11.2) [20]	0.098
P12	F/53	CML	Abnormal female chromosome 46,XX,t(9;22)(q34;q11.2) [22]	0.036
P13	F/82	CML	Abnormal female chromosome 46,XX,t(9;22)(q34;q11.2) [22]	0.057
P14	M/47	T-ALL	Normal male chromosome	0.070

Abbreviations: AML, acute myeloid leukemia; CML, chronic myeloid leukemia; T-ALL, T-cell acute lymphoblastic leukemia; FAB, French-American-British classification; F, female; M, male.
